# Comparative Histological Evaluation of Collagen Matrix Architectures for Soft Tissue Augmentation in the Oral Cavity: A Preclinical Canine Model

**DOI:** 10.3390/bioengineering13060662

**Published:** 2026-06-05

**Authors:** Hana Shah, Nicholas J. Iglesias, Sara E. Munkwitz, Blaire V. Slavin, Zachary M. Stauber, Quinn T. Ehlen, Vasudev Vivekanand Nayak, Seth R. Thaller, Lukasz Witek, Paulo G. Coelho

**Affiliations:** 1 University of Miami Miller School of Medicine, Miami, FL 33136, USA; 2 Florida International University Herbert Wertheim College of Medicine, Miami, FL 33199, USA; 3 DeWitt Daughtry Family Department of Surgery, University of Miami Miller School of Medicine, Miami, FL 33136, USA; 4 Dermatology and Plastic Surgery Institute, Cleveland Clinic, Cleveland, OH 44195, USA; 5 Department of Biochemistry and Molecular Biology, University of Miami Miller School of Medicine, Miami, FL 33136, USA; 6 Dr. John T. Macdonald Foundation Biomedical Nanotechnology Institute (BioNIUM), University of Miami, Miami, FL 33136, USA; 7 Biomaterials and Regenerative Biology Division, NYU College of Dentistry, New York, NY 10010, USA; 8 Department of Biomedical Engineering, NYU Tandon School of Engineering, Brooklyn, NY 11201, USA; 9 Hansjörg Wyss Department of Plastic Surgery, NYU Grossman School of Medicine, New York, NY 10016, USA; 10 Department of Oral and Maxillofacial Surgery, NYU College of Dentistry, New York, NY 10010, USA; 11 DeWitt Daughtry Family Department of Surgery, Division of Plastic Surgery, University of Miami Miller School of Medicine, Miami, FL 33136, USA; 12 Sylvester Comprehensive Cancer Center, University of Miami Miller School of Medicine, Miami, FL 33136, USA

**Keywords:** collagen matrix, soft tissue augmentation, xenograft, intraoral augmentation

## Abstract

Intraoral soft-tissue defects are traditionally managed with autogenous connective tissue grafts, though donor site morbidity has driven interest in xenogeneic collagen matrices as alternatives. However, the impact of matrix architecture on soft-tissue integration remains poorly understood. This study compared soft-tissue responses to a sheet-form collagen matrix (ShCM) and a spongy collagen matrix (SpCM) placed beneath full-thickness flaps in a beagle mandibular defect model. Standardized bilateral defects were created in 23 skeletally mature female beagles. Defects were then assigned to serve as the negative control (sham) or were treated with porcine collagen matrix in (i) sheet form (ShCM) (Regenity Biosciences, Oakland, NJ, USA), or (ii) porous/spongy form (SpCM) (Fibro-Gide^®^, Geistlich Pharma North America, West Windsor Township, NJ, USA). The mandibular sites that received no surgical intervention served as positive controls. Experimental conditions were randomized and interpolated within each animal to minimize anatomical site bias and evaluated histologically at 4- (*n* = 7), 8- (*n* = 7), and 12-weeks (*n* = 9) postoperatively. Histologic sections were evaluated for matrix presence, inflammation, subepithelial healing, and matrix thickness. At 4 weeks, both matrices were present, though SpCM showed significantly higher inflammation scores (*p* = 0.013). By 8 weeks, ShCM demonstrated greater resorption (*p* = 0.003) alongside an organized collagen layer with fibroblasts and new microvessels, while SpCM remained thick and porous, with a persistent fibrous capsule and elevated inflammation versus both ShCM (*p* = 0.002) and sham (*p* = 0.009). At 12 weeks, inflammation declined and subepithelial healing improved similarly across matrix groups. These findings suggest matrix architecture influences soft-tissue healing outcomes in the oral cavity. Sheet-form matrices may be preferable where biocompatibility and predictable integration are priorities, while spongy matrices may better support long-term space maintenance and tissue ingrowth. However, clinical studies are needed to confirm these translational implications.

## 1. Introduction

The craniofacial region consists of a complex interplay between hard and soft tissue structures. Collectively, these support mastication, speech, and overall function [1]. Defects affecting the soft tissue are highly prevalent, but current reconstructive approaches fall short of achieving predictable and stable long-term healing outcomes [1]. Autogenous connective-tissue grafts remain the gold standard for soft-tissue augmentation. However, longer operative times, donor site morbidity, and increased postoperative pain associated with autograft harvesting have warranted exploration of non-autologous substitutes [2,3,4,5,6]. Collagen, a major extracellular matrix component, is widely incorporated into these regenerative modalities because of its biocompatibility, low immunogenicity, and capacity to support neovascularization [7]. Nevertheless, native collagen is limited by rapid resorption in vivo, limited mechanical stability, and postoperative shrinkage, all of which may compromise soft-tissue volume during healing [1].

To address these limitations, xenogeneic collagen matrices of porcine origin have been introduced as alternatives for soft-tissue augmentation given their favorable biocompatibility profiles and low reported antigenicity [8,9]. Commercially available collagen matrices can differ in their degree of crosslinking, thickness, and porosity, all of which influence degradation kinetics and host tissue integration [10]. Non-cross-linked matrices tend to favor rapid vascularization, cell infiltration, and remodeling; however, they resorb quickly [11]. In contrast, cross-linked matrices are better able to resist early collapse and exhibit slower resorption and delayed tissue turnover [12,13]. Thickness also plays an important role, as thicker three-dimensional (3D) matrices generally provide greater space maintenance than thinner counterparts [14]. Similarly, porosity influences matrix permeability and biologic incorporation, with larger interconnected pores facilitating fibrovascular ingrowth and nutrient diffusion, while denser architectures may provide greater structural integrity but limit cellular penetration [15]. Beyond these properties, collagen matrices may also differ in their overall internal organization. For example, some may incorporate a dense superficial layer overlying a porous substructure designed to facilitate epithelialization and promote keratinized tissue formation.

Clinical studies have demonstrated that collagen-based matrices can increase keratinized mucosa around dental implants while maintaining peri-implant health [9,16,17,18,19]. Because these materials are intended to support epithelial-driven remodeling, they are commonly used in conjunction with partial-thickness flap designs that preserve the periosteal vascular bed [9,20,21,22]. In contrast, they may also be placed beneath full-thickness flaps to create a protected subperiosteal environment that enhances space maintenance, and fibrovascular integration. Although porcine-derived collagen matrices can gradually release key growth factors involved in angiogenesis and remodeling, such as platelet-derived growth factor (PDGF) and fibroblast growth factor II (FGF-2), their structural architecture likely remains a major determinant of the healing outcome [23].

Despite increasing clinical use of porcine-derived collagen biomaterials, the extent to which matrix architecture independently influences soft-tissue healing behavior under standardized surgical conditions remains poorly understood. This study aimed to compare the soft-tissue response to a sheet-form porcine collagen matrix (ShCM) and a porous, sponge-like collagen matrix (SpCM) placed beneath full-thickness flaps in a beagle mandibular model. Given that this study did not aim to assess keratinized tissue formation, a full-thickness flap was used as the standardized surgical approach across all treatment groups to isolate the effect of matrix architecture on sub-epithelial healing outcomes. We hypothesized that ShCM would exhibit faster sub-epithelial healing and a lower inflammatory response, whereas SpCM would demonstrate greater persistence and space-maintaining capacity during healing. Ultimately, these findings may guide clinicians in selecting the most appropriate matrix architecture based on whether rapid integration, minimal inflammatory response, and/or sustained volume maintenance is the primary therapeutic goal.

## 2. Materials and Methods

### 2.1. Surgical Protocol

**Figure 1:**
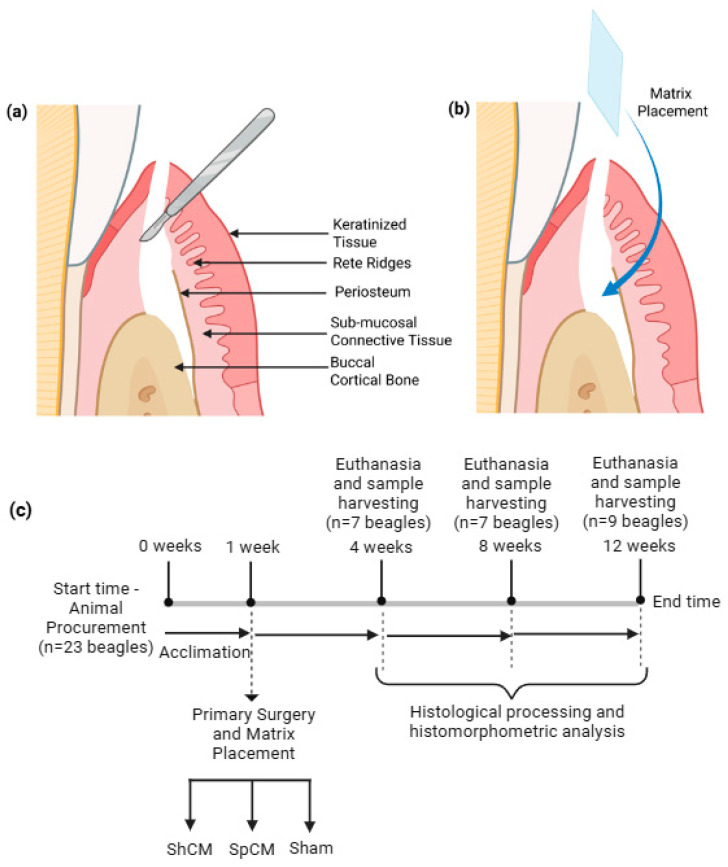
Schematic illustration of defect location, preparation, and matrix placement. (**a**) A full-thickness mucoperiosteal flap was elevated, and the defect was prepared by removing the submucosal connective tissue and periosteum to expose the underlying buccal cortical bone. (**b**) The assigned collagen matrix was placed within the prepared defect site before flap closure. (**c**) Study timeline. Created in Biorender. Vasudev Vivekanand Nayak. (2026). https://www.biorender.com/.

Experimental procedures were approved by the Institutional Animal Care and Use Committee of North American Science Associates, LLC (Coon Rapids, MN, USA) (protocol # IAN004-IS75). *N* = 23 female beagles were allowed to acclimate for ~1 week prior to surgical intervention. The animals were fasted overnight prior to surgery. All procedures were performed under general anesthesia and aseptic conditions. The animals were anesthetized with an intramuscular injection of midazolam (0.1–0.2 mg/kg) and butorphanol (0.05–0.1 mg/kg). During the procedure, anesthesia was maintained with inhaled isoflurane (2–5%) administered via an endotracheal tube and augmented with intravenous administration of propofol (2–8 mg/kg). Prior to defect creation, an additional bilateral caudal mandibular block with bupivacaine (0.5–2.0 mg/kg) was administered. The sites were prepared by creating a full-thickness flap. Defects [15 × 10 mm − length (anterior–posterior) × height (superior-inferior)] were created to remove the submucosal connective tissue and periosteum to expose the underlying mandibular bone bilaterally (Figure 1a).

Defects were then assigned to serve as the negative control (sham) or were treated with (i) porcine collagen matrix in sheet form (ShCM) (Regenity Biosciences, Oakland, NJ, USA) or (ii) porcine collagen matrix in porous or spongy form (SpCM) (Fibro-Gide^®^, Geistlich Pharma North America, West Windsor Township, NJ, USA) (Figure 1b). The mandibular sites that received no surgical intervention served as positive controls. Experimental conditions were randomized and interpolated within each animal to minimize anatomical site bias. Both matrices had an initial thickness of up to 3 mm. SpCM was selected as a comparator because it is a commercially available, volume-stable collagen matrix with prior preclinical and clinical use in soft tissue augmentation [7,24]. These studies support its use as a clinically relevant reference biomaterial against which the healing behavior of the sheet-form matrix could be compared. Defect sites were sutured using polytetrafluoroethylene (PTFE) sutures.

Post-operative analgesia was provided via intramuscular injections of buprenorphine (0.05 mg/kg), tramadol (4–10 mg/kg), carprofen (2.5–5.0 mg/kg), and cefpodoxime (5–10 mg/kg) as required. Food and water were provided ad libitum, and oral incisions were routinely monitored. Only the study surgeons (L.W. and P.G.C.) were aware of the group distributions through the course of the study. Euthanasia was performed at either 4 (*n* = 7), 8 (*n* = 7), or 12 weeks (*n* = 9) post-operatively. A larger cohort was assigned to the 12-week timepoint to account for potential subject attrition due to adverse effects or surgical complications over the extended follow-up period. The mandibles were harvested en bloc and immersed in 10% formalin for subsequent analyses. The study timeline is summarized in Figure 1c.

### 2.2. Histological Processing

**Figure 2:**
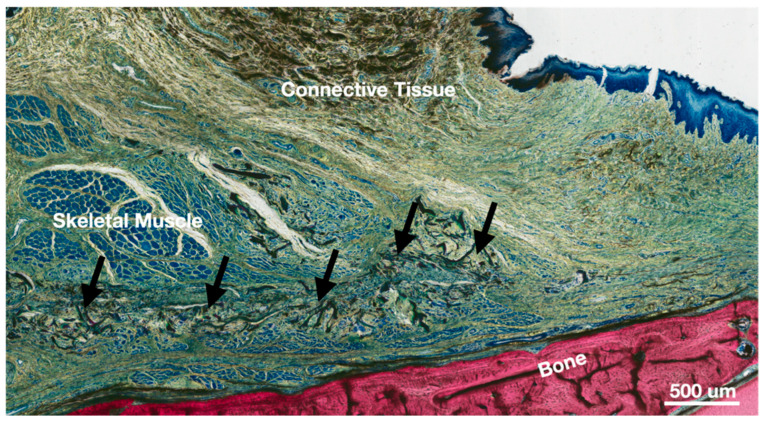
An example of a soft tissue defect repaired with a porcine-derived collagen matrix (black arrows) after 4 weeks of permitted healing. SVG stained bone and tooth structures in red, collagen fibers within connective tissue in blue-green, and skeletal muscle in blue.

The mandible samples were gradually dehydrated in a series of ethanol solutions ranging from 70 to 100% *v*/*v*. Following dehydration, the samples were immersed in methyl salicylate and subsequently embedded in a methacrylate-based resin according to a previously established protocol [1]. Embedded samples were cut into wafers (~300 μm thick) in the buccal–lingual orientation of the mandible using a precise, low-speed diamond wafering saw (Isomet 2000, Buehler Ltd., Lake Bluff, IL, USA). Wafers were glued to acrylic slides with a low-viscosity cyanoacrylate-based adhesive (Loctite 408, Henkel AG, Dusseldorf, Germany). After setting for 24 h, the mounted specimens were reduced to a final thickness of ~80 μm by means of grinding using a series of silicon carbide abrasive papers (400, 600, 800, and 1200 grit; Buehler Ltd., Lake Bluff, IL, USA) on a grinding/polishing machine (Metaserv 3000, Buehler Ltd., Lake Bluff, IL, USA) under copious irrigation. Subsequently, the samples were polished using a polishing suspension (1 µm MicroPolish^TM^, Buehler Ltd., Lake Bluff, IL, USA) and stained with Stevenel’s Blue (Thermo Fisher Scientific, Waltham, MA, USA) and Van Gieson’s Picro Fuchsin (SVG) (American MasterTech Scientific, Lodi, CA, USA). This staining combination permitted the differentiation between the soft, connective, and mineralized tissue structures (Figure 2). Slides were digitally scanned via an automated slide scanning system and specialized computer software (Aperio CS2 and ImageScope v12.4.6, Leica Biosystems, Deer Park, IL, USA). Histological slides were also analyzed using a light microscope (DM2500M, Leica Biosystems, Deer Park, IL, USA) equipped with a polarizer and camera module (Flexacam C5, Leica Biosystems, Deer Park, IL, USA).

### 2.3. Semiquantitative Analysis

**Figure 3:**
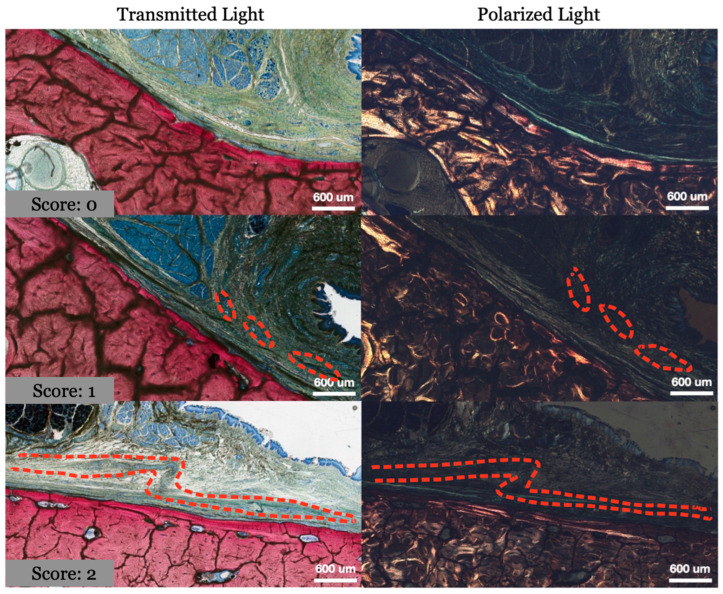
Matrix presence was evaluated and scored by visualization under transmitted and polarized light microscopy. A score of 0, 1, and 2 was defined by matrix absence, partial, and full presence, respectively, indicated by dashed red splines on representative histomicrographs.

**Figure 4:**
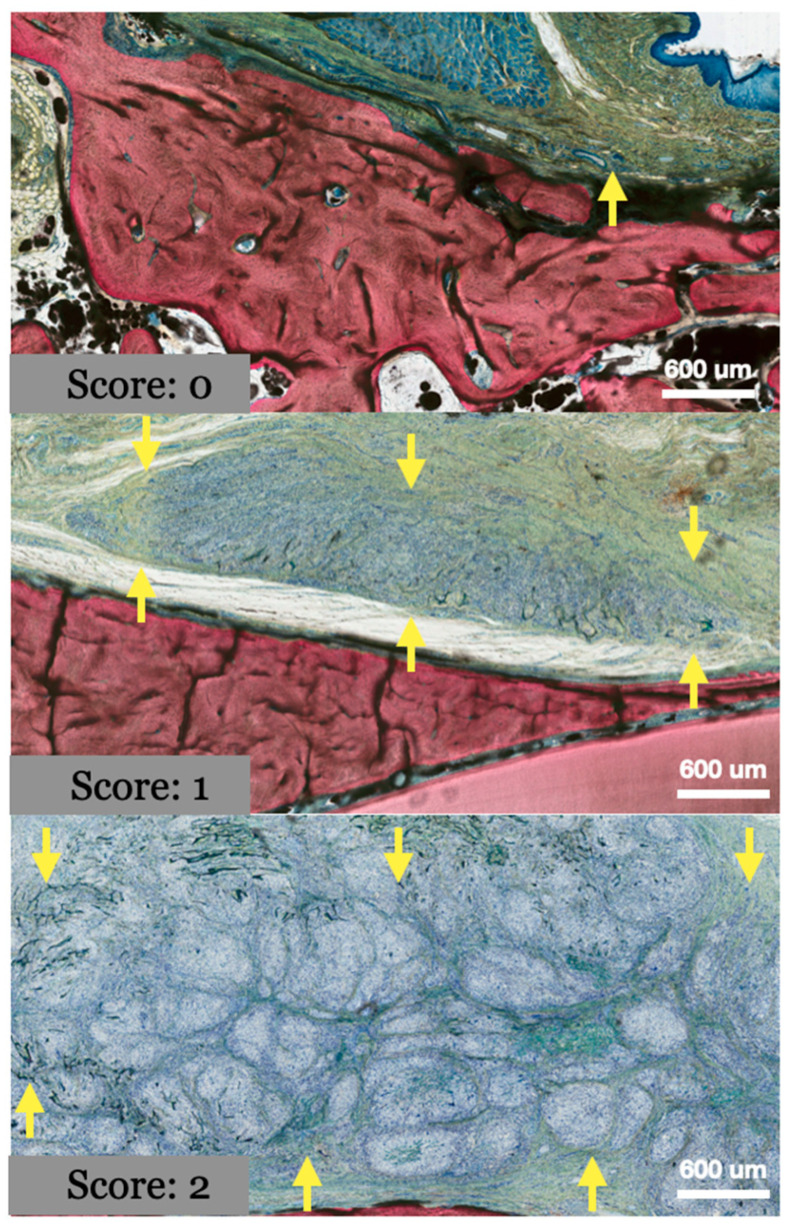
Inflammation was evaluated and scored by visualization under transmitted light microscopy. A score of 0, 1, and 2 was defined by no/minimal, mild and high inflammation, respectively, indicated by yellow arrows on representative histomicrographs.

**Figure 5:**
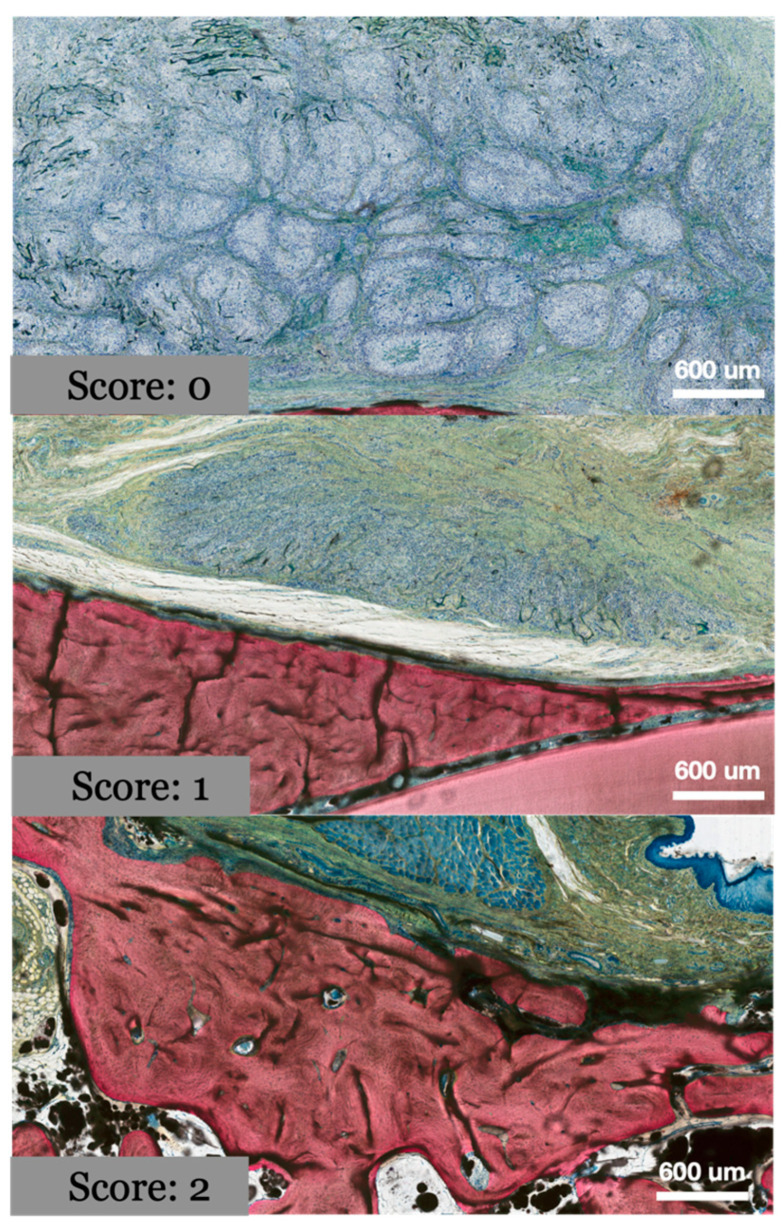
Subepithelial healing was evaluated and scored by visualization under transmitted light microscopy.

Matrix presence (Table 1, Figure 3), inflammation (Table 2, Figure 4), and subepithelial healing (Table 3, Figure 5) at each treated defect site were evaluated semi-quantitatively using a scale ranging from 0 to 2. Histologic specimens were analyzed by a single, trained and experienced investigator who was blinded to treatment groups and study time points.

### 2.4. Quantitative Analysis

**Figure 6:**
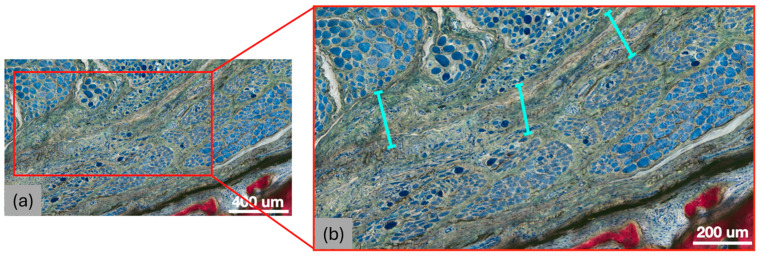
(**a**) Low magnification representative histomicrograph indicating the region of interest (defect site with matrix presence) in red box, and (**b**) high magnification representative histomicrograph detailing the 3 distinct locations along the buccal wall used to quantify matrix thickness (cyan annotations).

Histological slides were scanned and assessed for matrix thickness within the defect site using Imagescope v12.4.6 (Leica Biosystems, Deer Park, IL, USA). Matrix thickness (μm) was measured at 3 distinct locations along the length of the matrix placed against the buccal wall (Figure 6) at the 3 healing time points.

### 2.5. Sample Size Calculation

Subepithelial healing was considered the primary outcome variable, and the sample size was determined based on an a priori power analysis using G*Power v3.1.9.7 (Heinrich-Heine-Universität Düsseldorf, Düsseldorf, Germany). Assuming a probability of a false positive (*α*) = 0.05, power = 0.80, and effect size = 0.5, a total of *n* = 69 samples were required to address the study hypothesis. Mature beagles were selected as the animal model due to their large mandibular anatomy, and as they are well established for evaluating oral regenerative biomaterials given their clinically relevant mucosal architecture and wound healing characteristics [25,26,27,28]. Therefore, *n* = 23 skeletally mature beagles were acquired, which allowed for nesting of groups within subjects.

### 2.6. Statistical Methods

Statistical analysis was performed using the Kruskal–Wallis (KW) non-parametric test using IBM SPSS v29 (IBM Corp., Armonk, NY, USA) with post hoc pairwise comparisons conducted within the KW framework, where applicable. Data is presented as medians and interquartile ranges (IQR), with *p* < 0.05 indicating statistical significance.

## 3. Results

### 3.1. Qualitative Histologic Findings

**Figure 7:**
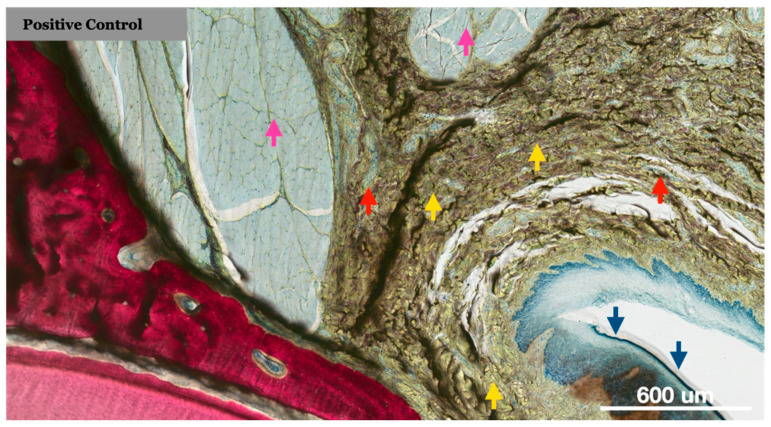
Histomicrographs of the epithelium and subepithelium in the positive control group. Blue arrows highlight stratified squamous epithelium, red arrows point to arterioles, yellow arrows point to collagen fibers and native lamina propria structure and pink arrows showcase skeletal muscle.

Subepithelial changes were investigated as a function of time and matrix. The subepithelial morphology from the positive control samples served as the baseline. Positive controls presented a dense layer of connective tissue (lamina propria) beneath a stratified squamous epithelium (Figure 7). The superficial connective tissue, or papillary layer, consisted of loosely arranged collagen fibers that formed papillary projections between undulating epithelial rete ridges. This transitioned into the reticular layer, densely concentrated collagen fibers lying parallel with underlying alveolar bone. Interspersed within these subepithelial layers, fibroblasts, arterioles, and skeletal muscle were visible.

**Figure 8:**
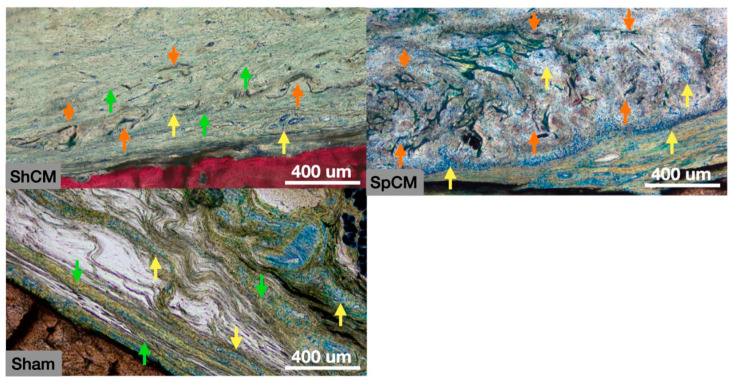
Inflammation (yellow arrows), matrix presence (orange arrows), and matrix degradation with soft tissue healing (green arrows) for the ShCM, SpCM and Sham groups after 4 weeks of permitted healing.

Histologic sections at the early healing time point (4 weeks) revealed evidence of a foreign body reaction within full-thickness defects (Figure 8). It is important to note that ShCM exhibited a thin, sheet-like structure. In contrast, SpCM appeared thicker, with a porous, sponge-like morphology. Degree of inflammation and matrix presence varied based upon treatment group. A high degree of inflammatory cells, macrophages and/or foreign body giant cells was noted to surround SpCM within a well-defined fibrous capsule. In contrast, ShCM and sham had minimal to no inflammatory cell presence. Full to partial matrix presence was seen with SpCM, while resorption was evident in ShCM with intimate integration between the matrix and newly regenerating soft tissue.

**Figure 9:**
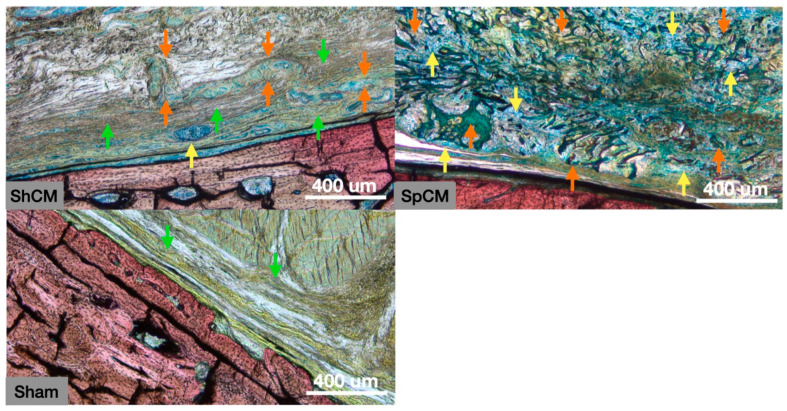
Inflammation (yellow arrows), matrix presence (orange arrows), and matrix degradation with soft tissue remodeling (green arrows) for the ShCM, SpCM and Sham groups after 8 weeks of permitted healing.

In comparison to 4 weeks, an overall reduction in inflammatory content was visualized within all treatment groups, except SpCM, at the 8-week time point (Figure 9). SpCM exhibited a similar inflammatory cell presence as seen at 4 weeks, although a larger population of foreign body giant cells was present in place of macrophages within a persistent fibrous capsule. In addition, SpCM degradation was not discernible between 4 and 8 weeks. On the other hand, ShCM demonstrated significant resorption at this timepoint relative to 4 weeks, which now resembled a compact layer of organized collagen fibers and interspersed fibroblasts. This indicated soft tissue remodeling at the defect site. Small, new blood vessels were also well visualized in the soft tissue surrounding ShCM; however, these were not seen in SpCM. The sham group depicted no major morphological differences in comparison to the early healing time point.

**Figure 10:**
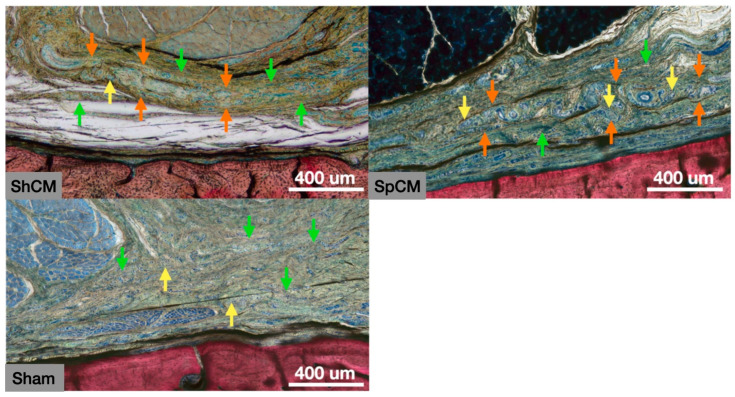
Inflammation (yellow arrows), matrix presence (orange arrows), and matrix degradation with soft tissue remodeling (green arrows) for the ShCM, SpCM and Sham groups after 12 weeks of permitted healing.

All experimental groups at the extended healing time point (12 weeks) demonstrated minimal inflammatory presence relative to the earlier time points (Figure 10). Of note, SpCM presented a reduction in engrossing inflammatory cells between 8 and 12 weeks. Regarding matrix presence, SpCM continued to appear thick and apparent. Defects treated with ShCM presented greater matrix resorption and gradual return to native soft tissue structure. Nonetheless, a higher degree of neovascularization was evident among all groups at this timepoint within the defect site.

### 3.2. Semi-Quantitative Histologic Findings

**Figure 11:**
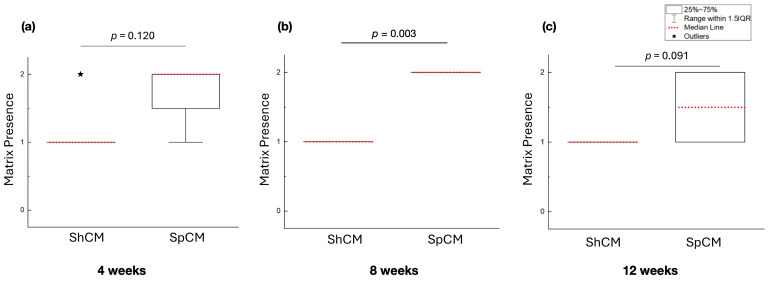
Ranked matrix presence between treatment groups at (**a**) 4 weeks, (**b**) 8 weeks, and (**c**) 12 weeks of permitted healing. *p* < 0.05 is statistically significant.

**Figure 12:**
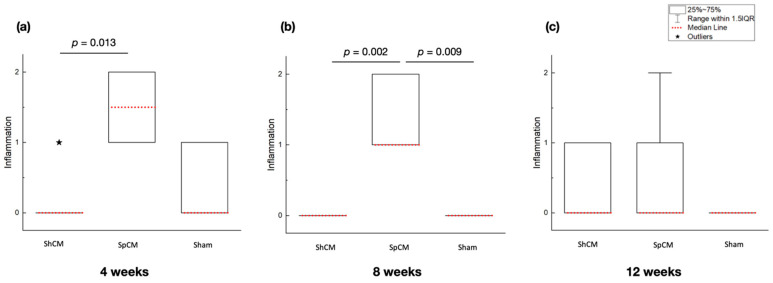
Ranked inflammation between treatment groups at (**a**) 4 weeks, (**b**) 8 weeks, and (**c**) 12 weeks of permitted healing. *p* < 0.05 is statistically significant.

Ranked matrix presence, inflammation, and subepithelial healing values are summarized in Table 4. At 4 weeks (Figure 11a), SpCM showed similar presence to ShCM (*p* = 0.120). At 8 weeks (Figure 11b), ShCM presented a greater degree of resorption relative to SpCM (*p* = 0.003). This was in agreement with the aforementioned qualitative evaluation of the histomicrographs. A similar trend in matrix presence was discerned at 12 weeks (Figure 11c) with a reduction in residual matrix within the defect site in both treatment groups. SpCM presented significantly higher inflammation than ShCM at 4 weeks (*p* = 0.013) (Figure 12a). No statistically significant differences in inflammation were observed between ShCM and sham at the 4-week time point. Inflammation among all groups was reduced at 8 weeks, relative to 4 weeks (Figure 12b). Of note, SpCM demonstrated significantly higher inflammatory content compared to ShCM (*p* = 0.002), and sham (*p* = 0.009). Inflammation among all groups was lower at the 12-week time point (compared to that at 8 weeks) (Figure 12c); however, no statistical differences were observed between any of the treatment groups at this extended healing time point.

**Figure 13:**
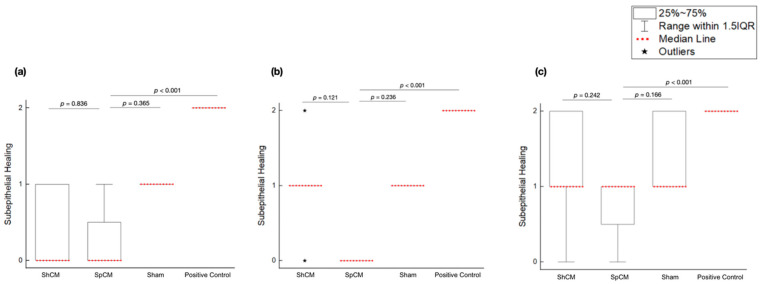
Ranked subepithelial healing between treatment groups at (**a**) 4 weeks, (**b**) 8 weeks, and (**c**) 12 weeks of permitted healing, compared to SpCM. *p* < 0.05 is statistically significant.

**Figure 14:**
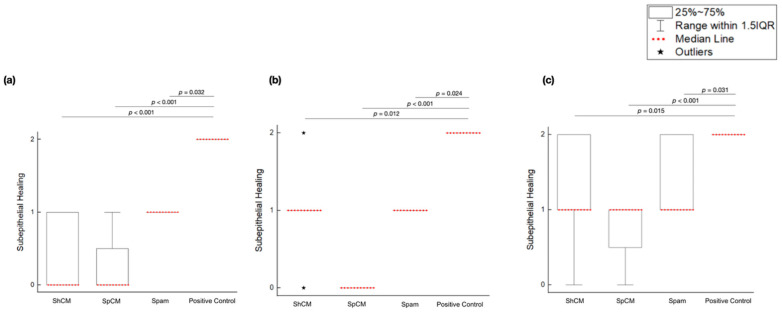
Ranked subepithelial healing between treatment groups at (**a**) 4 weeks, (**b**) 8 weeks, and (**c**) 12 weeks of permitted healing, compared to positive control. *p* < 0.05 is statistically significant.

Irrespective of the time point of evaluation, subepithelial healing was statistically homogenous between SpCM relative to the ShCM (*p* = 0.836, *p* = 0.121, and *p* = 0.242 at 4, 8 and 12 weeks, respectively) and sham (*p* = 0.365, *p* = 0.236, and *p* = 0.166 at 4, 8 and 12 weeks, respectively) (Figure 13). As a reference, the positive control group presented the unoperated tissue morphology expected within the region of interest. Irrespective of the time point of evaluation, all groups that received treatment (with or without matrix) presented significantly lower subepithelial healing relative to the positive control (*p* < 0.05) (Figure 14).

### 3.3. Quantitative Histologic Findings

**Figure 15:**
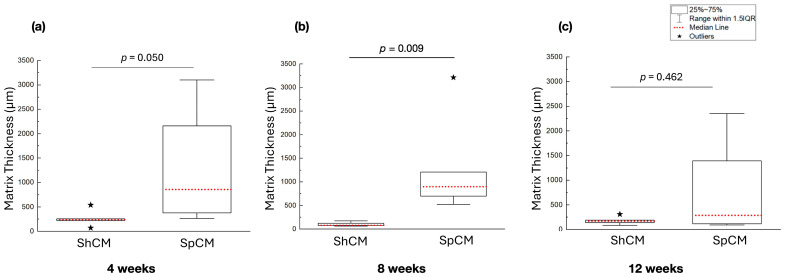
Matrix thickness (μm) of the various treatment groups at (**a**) 4 weeks, (**b**) 8 weeks, and (**c**) 12 weeks of permitted healing. *p* < 0.05 is statistically significant.

Matrix thickness values are summarized in Table 5. At 4 weeks (Figure 15a), there was no significant difference in matrix thickness between ShCM and SpCM. At 8 weeks (Figure 15b), matrix thickness values were reduced in both groups, and SpCM was significantly thicker than ShCM (*p =* 0.009). At the extended healing time point, 12 weeks (Figure 15c), no significant differences in thickness values were observed (*p* = 0.462). However, across timepoints, SpCM presented wider IQRs of matrix thickness values likely reflecting heterogeneity of the spongy architecture in contrast to ShCM—which demonstrated consistently narrow IQRs, indicative of a uniform matrix thickness.

## 4. Discussion

This study compared the soft-tissue response of a porcine collagen matrix in sheet form (ShCM) to a porous, sponge-like collagen matrix (SpCM) in a beagle mandibular soft tissue defect model. Histologically, SpCM defects exhibited a more pronounced foreign body reaction with macrophage and foreign body giant cell accumulation within the fibrous capsule at 4 and 8 weeks, only partially resolving by 12 weeks. This pattern is consistent with typical foreign body reaction to implanted biomaterials, in which the material surface becomes a biological interface wherein the material physiochemical properties dictate protein adsorption, monocyte adhesion, macrophage differentiation, and foreign body giant cell formation [2]. On the other hand, ShCM-treated sites revealed minimal inflammatory infiltrates across all time points and were indistinguishable from sham on semi-quantitative scoring. These findings suggest that differences in both matrix architecture and surface topography likely contributed to the direct host responses observed, with the sheet-like configuration of ShCM favoring a more limited inflammatory reaction than the porous SpCM [3]. The foreign body reaction progresses towards fibrotic capsule formation, which can mature and stiffen around the implanted material, hindering integration and predisposing to adverse clinical events [4]. As such, the reduced inflammatory reaction seen in ShCM may highlight a more favorable host-biomaterial interaction.

ShCM underwent earlier resorption and progressive integration with surrounding connective tissue, whereas SpCM remained thicker, more porous, and structurally persistent over time. At 4 weeks, both materials were discernible within the defect, but by 8 weeks, semi-quantitative scoring showed significantly greater resorption of ShCM compared to SpCM, with a marked reduction in ShCM thickness. This finding aligns with a prior study that collagen sheets undergo rapid, surface-mediated degradation, whereas porous, sponge-like matrices prolong foreign body giant cell-mediated degradation and encapsulation because their greater surface area allows for protein adsorption and macrophage attachment [5]. From a clinical standpoint, the present findings suggest that ShCM follows a controlled resorption pattern compatible with the timeline of soft tissue healing [6], while SpCM behaves more as a long-lasting volumetric scaffold [7]. The increase in median ShCM thickness at 12 weeks relative to 8 weeks likely reflects measurement limitations inherent to late-stage histologic assessment. In brief, this can be attributed to the challenge of distinguishing residual matrix from re-modeled collagenous tissue once the matrix becomes more integrated with the surrounding host connective tissue [5,6].

The selection of SpCM as a comparator was intentional. Thoma et al. investigated a volume-stable three-dimensional collagen matrix as a substitute for connective tissue grafting for ridge contour augmentation [24]. In a five-year follow-up study, they found that the collagen matrix maintained peri-implant health, presented with favorable aesthetics, and showed no significant differences in buccal mucosal thickness or ridge contour changes relative to the connective tissue graft [7,24]. While SpCM may be beneficial in cases of severe peri-implant deficiency or horizontal ridge defects, its excessive persistence may promote sustained foreign body reaction and delay restoration of native tissue architecture [8].

ShCM-treated defects developed a more compact and organized subepithelial collagen layer with fibroblasts and newly formed microvessels, while SpCM sites showed more persistent matrix remnants and delayed tissue maturation. As expected, positive control sites displayed stratified squamous epithelium over the lamina propria with a mature collagen network and interspersed vasculature, muscle fibers, and fibroblasts. Initially, treated sites showed lower subepithelial healing scores compared to the positive control, likely reflecting the disruption of native structure following defect creation [9]. This pattern is consistent with prior observations that the underlying connective tissue influences the characteristics of the overlaying oral epithelium [9,29,30]. Surgical injury leads to the temporary loss of native, keratinization-inducing sub-epithelial connective tissue, with the defect initially filled by granulation tissue [9,31]. As the granulation tissue matures, the epithelium shifts towards a more keratinized phenotype with subepithelial scores rising after approximately 7–14 days [9,29,31,32]. In the present study, ShCM-treated defects developed a compact, organized collagen layer with fibroblasts and newly formed microvessels. In contrast, SpCM sites revealed delayed recovery with persistent matrix remnants and evidence of ongoing remodeling. Rather than suggesting that a matrix is unnecessary, this pattern may support the idea that sheet-like matrices can function as transient scaffolds that may be more readily replaced by host-derived connective tissue, while spongy matrices may require more time to achieve comparable soft-tissue maturation [16].

Moreover, ShCM showed closer integration with the surrounding connective tissue and greater evidence of microvessels in remodeling zones, whereas SpCM remained associated with a more persistent fibrous capsule. Prior studies have shown that adequate vascular ingrowth is important for nutrient delivery, waste removal, cellular migration, and successful bone and soft-tissue regeneration [17]. The integration of ShCM with the surrounding connective tissue and presence of microvessels in remodeling zones suggests that the sheet architecture facilitates early angiogenic responses [18,19]. For instance, studies have noted that oxygen supply is limited to a diffusion distance of ~150–200 μm from the nearest capillary, emphasizing the importance of short diffusion paths and reduced barrier thickness for adequate cell survival [18,19]. Limited diffusion distances and interconnected porosities favor homogenous vascular infiltration of collagen-based devices, thus, accelerating their integration and minimizing chronic foreign body giant cell recruitment [10]. On the other hand, the persistent fibrous capsule and spongy matrix (such as SpCM)-treated sites likely inhibit rapid vascular integration, aligning with reports that a thick, fibrous capsule isolates biomaterials from the surrounding tissue environment and impairs tissue repair [11,12,13]. Nevertheless, the spongy architecture of SpCM may be ideal for blood clot stabilization and maintenance of space for tissue ingrowth in oral regenerative procedures [21,23,33].

The findings from this study align with the literature supporting xenogeneic collagen matrices as safe alternatives to connective tissue grafts [20,33]. While they provide less soft tissue gain than connective tissue grafts, xenogeneic collagen matrices are valuable in cases where patient comfort is prioritized and can achieve clinically relevant results [22]. Previous studies have evaluated soft-tissue augmentation in partial-thickness flap designs [9]; however, the current study specifically looked at ShCM and SpCM beneath a full-thickness flap. While this approach was intentionally used to isolate the effect of matrix architecture as the primary variable of interest, it precludes conclusions about how flap type interacts with matrix performance. This is a meaningful gap as clinical data has shown that partial-thickness flap designs increase soft tissue thickness and result in greater keratinized tissue width relative to full-thickness approaches [14,15]. In a full-thickness setting, the choice of biomaterial becomes important because the periosteal blood supply is temporarily disrupted and must be re-established in the augmented region. The present data shows that not all collagen-based devices are equivalent [16]. Subtle differences in structural design (i.e., sheet vs. sponge) can influence host response and clinical performance. These observations have direct implications for clinical practice. Sheet-form collagen matrices appear to be well suited for cases where rapid integration, minimal chronic inflammation, and restoration of native-like subepithelial architecture are prioritized. Clinical trials with xenogeneic collagen matrices have shown increases in keratinized tissue and soft tissue thickness, with good integration, lower pain and operative time compared to connective tissue grafts [34]. In contrast, spongy, volume-stable collagen matrices remain beneficial in cases that require sustained space maintenance or bulk augmentation, like buccal soft tissue thickening or contour augmentations [24,35]. However, their selection should be approached with caution given their increased inflammatory profile and slower remodeling.

While the preclinical beagle model is highly translational for oral and craniofacial applications, it still may not fully replicate human soft tissue biology or functional loading [36]. Additionally, the evaluation of soft-tissue healing was restricted to 12 weeks, which captures proliferative and early remodeling phases of wound healing, necessitating longer follow ups [37]. Direct volumetric measurement of soft tissue gain was not performed, and all conclusions regarding soft tissue augmentation rely on the two-dimensional (2D) histomicrographs that provide information pertaining to tissue quality and integration rather than volumetric soft tissue gain. Future work should therefore incorporate 3D volumetric outcome measures to directly assess the soft tissue augmentation achieved by these matrix architectures. Examining cytokine expression and angiogenic signaling in response to sheet versus spongy collagen matrix architectures could also provide valuable insights into the biological pathways driving the observed differences in inflammation and remodeling. The semi-quantitative scoring system also introduces observer subjectivity. Although the evaluator was blinded to treatment group and healing interval assignment, formal intra-reliability testing was not performed, and some degree of observer-dependent bias cannot be excluded.

Moreover, only two porcine collagen matrices with distinct architectures were examined. As such, given the heterogeneity in collagen source, cross-linking chemistry, and porosity of commercially available xenogeneic matrices, these results cannot be generalized to all xenogeneic collagen-based matrices. The present study did not include direct physicochemical characterization of matrix architecture or surface properties, such as pore size distribution, surface roughness, or surface chemistry. Therefore, the observed differences should be interpreted as histologic and biologic responses associated with the tested biomaterials rather than as direct measurements of the structural parameters responsible for those responses. Nonetheless, previous studies have shown that collagen matrices can differ in these characteristics depending on tissue origin and manufacturing method, supporting the possibility that such features contributed to the host responses observed in the present study [38,39]. Ultimately, clinical trials evaluating patient-reported outcomes, aesthetic satisfaction, and long-term soft tissue stability are warranted to translate these preclinical findings into evidence-based guidelines for matrix selection.

## Figures and Tables

**Table 1:** Ranking scale used to measure matrix presence in various treatment groups across timepoints.

Matrix Presence	Description
0	No matrix presence
1	Partial matrix presence
2	Full matrix presence

**Table 2:** Ranking scale used to measure inflammation in various treatment groups across timepoints.

Inflammation	Description
0	No/minimal inflammation
1	Mild inflammation
2	High inflammation

**Table 3:** Ranking scale used to measure subepithelial healing in various treatment groups across timepoints.

Ranked Subepithelial Healing Scale	Description
0	Poor degree of subepithelial healing (high inflammation and loosely organized collagen fibers relative to positive control)
1	Moderate degree of subepithelial tissue healing (mild inflammation and increasingly compact layer of organized collagen fibers relative to positive control)
2	High degree of subepithelial tissue healing (minimal inflammation, highly integrated matrix with neovascularization, and resemblance to native lamina propria of positive control)

**Table 4:** Descriptives of semi-quantitative matrix presence, inflammation, and ranked subepithelial healing at 4, 8, and 12 weeks.

	Group	Median (IQR)		
		4 Weeks	8 Weeks	12 Weeks
**Matrix Presence**	ShCM	1 (0)	1 (0)	1 (0)
	SpCM	2 (0.5)	2 (0)	1.5 (1)
**Inflammation**	ShCM	0 (0)	0 (0)	0 (1)
	SpCM	1.5 (1)	1 (1)	0 (1)
	Sham	0 (1)	0 (0)	0 (0)
**Ranked Subepithelial Healing Scale**	ShCM	0 (1)	1 (0)	1 (1)
	SpCM	0 (0.5)	0 (0)	1 (0.5)
	Sham	1 (0)	1 (0)	1 (1)

**Table 5:** Descriptives of quantitative matrix thickness at 4, 8, and 12 weeks.

	Median (IQR) (Units in μm)		
Group	4 Weeks	8 Weeks	12 Weeks
ShCM	238.57 (35.5)	78.93 (46.04)	169.73 (50.24)
SpCM	856.65 (1784.19)	896.70 (511.8)	286.30 (1276.82)

## Data Availability

The raw data supporting the conclusions of this article will be made available by the authors on request.
